# Context-sensitive decrement times for inhaled anesthetics in obese patients explored with Gas Man®

**DOI:** 10.1007/s10877-020-00477-z

**Published:** 2020-02-17

**Authors:** Jonas Weber, Johannes Schmidt, Steffen Wirth, Stefan Schumann, James H. Philip, Leopold H. J. Eberhart

**Affiliations:** 1 Department of Anesthesiology and Critical Care, Medical Center – University of Freiburg, Faculty of Medicine, University of Freiburg, Freiburg, Germany; 2 Department of Anesthesiology, Perioperative and Pain Medicine, Brigham and Women’s Hospital, Harvard Medical School, Boston, MA 02115 USA; 3 Department of Anaesthesiology and Intensive Care, Philipps-Universität Marburg, Marburg, Germany

**Keywords:** Anesthesia uptake and distribution, Inhalation anesthesia, Computer simulation, Pharmacokinetics, Physiology based model, Anesthesia decision support tools, End-tidal control delivery machines

## Abstract

Anesthesia care providers and anesthesia decision support tools use mathematical pharmacokinetic models to control delivery and especially removal of anesthetics from the patient’s body. However, these models are not able to reflect alterations in pharmacokinetics of volatile anesthetics caused by obesity. The primary aim of this study was to refine those models for obese patients. To investigate the effects of obesity on the elimination of desflurane, isoflurane and sevoflurane for various anesthesia durations, the Gas Man® computer simulation software was used. Four different models simulating patients with weights of 70 kg, 100 kg, 125 kg and 150 kg were constructed by increasing fat weight to the standard 70 kg model. For each modelled patient condition, the vaporizer was set to reach quickly and then maintain an alveolar concentration of 1.0 minimum alveolar concentration (MAC). Subsequently, the circuit was switched to an open (non-rebreathing) circuit model, the inspiratory anesthetic concentration was set to 0 and the time to the anesthetic decrements by 67% (awakening times), 90% (recovery times) and 95% (resolution times) in the vessel-rich tissue compartment including highly perfused tissue of the central nervous system were determined. Awakening times did not differ greatly between the simulation models. After volatile anesthesia with sevoflurane and isoflurane, awakening times were lower in the more obese simulation models. With increasing obesity, recovery and resolution times were higher. The additional adipose tissue in obese simulation models did not prolong awakening times and thus may act more like a sink for volatile anesthetics. The results of these simulations should be validated by comparing the elimination of volatile anesthetics in obese patients with data from our simulation models.

## Introduction

According to the World Health Organization, worldwide more than 1.9 billion adults were overweight and 41 million children (< 5 years) were obese in 2016 [1]. The excess fat tissue as well as the increase in cardiac output (CO), compared with lean body mass models, lead to changes in distribution, binding and most importantly elimination of many drugs in obese patients. Due to these pathophysiological alterations of pharmacokinetics, anesthesiologists are often confronted with difficulties in drug dosage in obese patients [2–5].

The lower blood-gas partition coefficient of modern volatile anesthetics, like desflurane and sevoflurane, provide more accelerated elimination compared to older ones [6–8]. Nevertheless, physicochemical differences exist also between the modern agents. For example, isoflurane has a higher fat/gas partition coefficient (64.2 ± 12.3) [9] than desflurane (12.0 ± 2.0) [10] and sevoflurane (34.0 ± 6.0) [9]. In accordance with the higher tissue/blood partition coefficients of sevoflurane, compared to isoflurane and desflurane, Casati et al. could demonstrate that after short surgical procedures, sevoflurane provides a more rapid elimination in morbidly obese patients than isoflurane [11].

Eger et al. characterized the factors governing volatile anesthetic pharmacokinetics theoretically. They described the blood flow and blood/gas partition coefficient as primary determinant of the volatile anesthetics’ dispersion and the tissue volume and tissue/gas partition coefficients as primary determinants of the size of the tissue depots [12]. It follows that the increased fat volume in obese patients increases the potential tissue depots. However, based on their theoretical observations on the comparable long time constants of fat tissue, numerous clinical studies [11, 13–16] and the comparable low fat perfusion in obese patients [17], Eger et al. concluded that obesity might not influence awakening materially [12].

As part of modern anesthetic drug administration strategies, anesthesia decision support tools like SmartPilot® (Dräger Medical, Lübeck, Germany) and Navigator® (GE Healthcare, Helsinki, Finland) allow prediction and display of effect site concentrations of anesthetics by using pharmacokinetic models and thereby help clinicians to adjust vaporizer and fresh gas flow [18–20]. Because of a lack in theoretical and clinical data to characterize the alterations of pharmacokinetics of volatile anesthetics in obese patients, these systems may have errors in their prediction. End-tidal control delivery machines like Zeus® (Dräger Medical), Aisys EtControl™ (GE Healthcare, Madison, WI, USA) and the FLOW-i (Maquet, Getinge AB, Getinge, Sweden) adjust the vaporizer output and fresh gas flow (FGF) automatically to reach and maintain a previously set end-tidal volatile anesthetics’ concentration [21–23] and will be less or not-at-all affected by model errors.

The primary hypothesis of our study was that the obesity-associated increase in tissue volumes, flows and CO delays the wash-out kinetics of desflurane, sevoflurane and isoflurane in a pharmacokinetic simulation model for volatile anesthetics. We further hypothesized that this pharmacokinetic model can be used to validate the theoretical observations by Eger et al. [12]. To test these hypotheses we used the existing Gas Man simulation software (version 4.2, retrieved from: www.medmansimulations.org) to construct mathematical physiology-based obese pharmacokinetic simulation models.

## Methods

To study uptake, distribution and elimination of inhalational anesthetics, the Gas Man simulation software has been used extensively [24–28]. This research report is based on simulation data and does not involve human subjects, either directly or indirectly. Thus, review by an Institutional Review Board was not required.

**Fig. 1:**
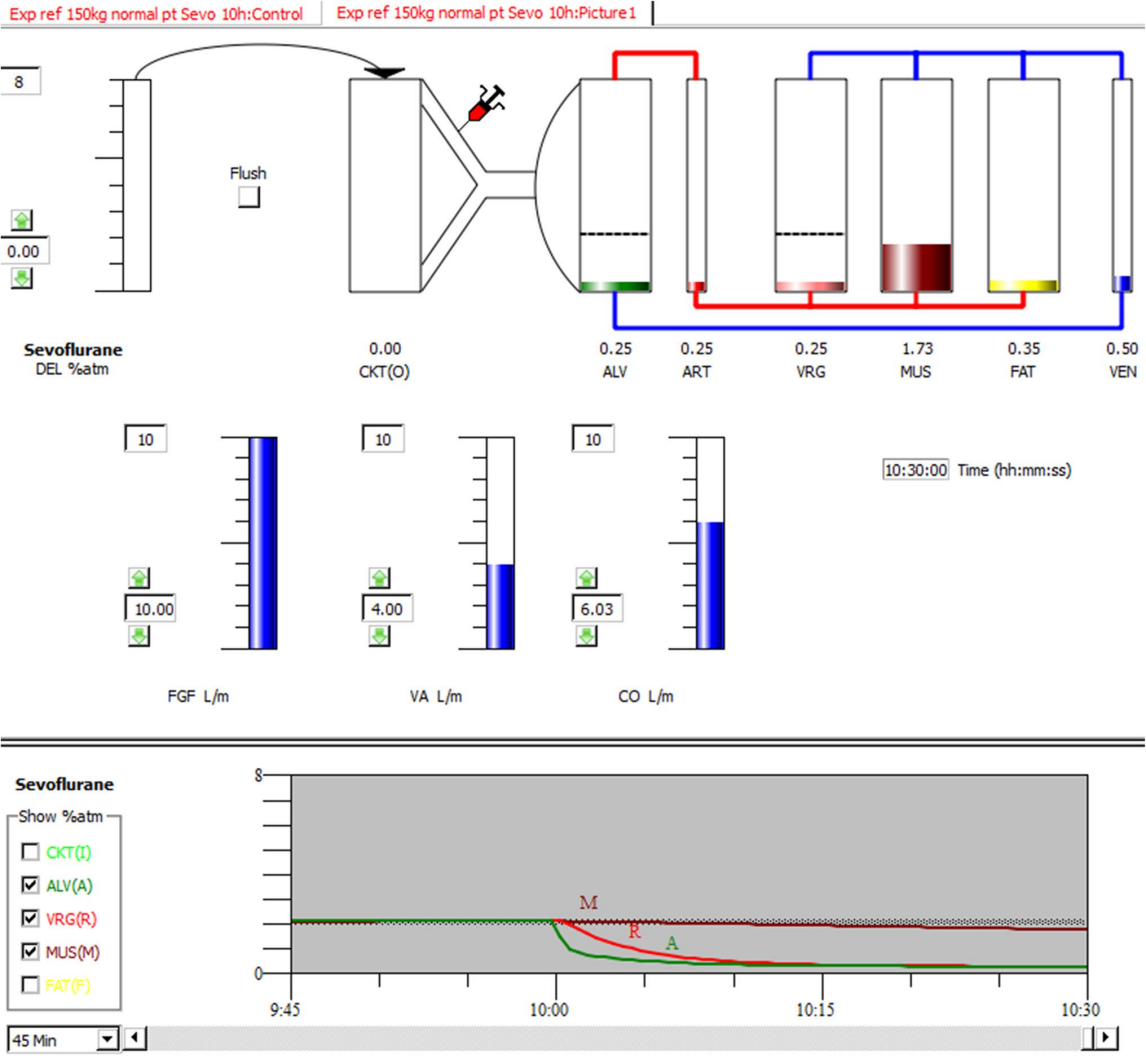
Gas Man Picture (top) and Graph (bottom) after 10 h of sevoflurane administration of 1.0 MAC expiratory. DEL, delivered sevoflurane partial pressure; CKT, circuit; ALV, alveolar; ART, arterial blood; VRG, vessel-rich-group; MUS, muscular; FAT, fat; VEN, venous blood; FGF, fresh gas flow; VA, alveolar ventilation; CO, cardiac output. The Gas Man Picture shows the actual duration of simulated administration (10 h and 30 min), the anesthetic tension in the different model compartments and some of the possible physiologic adaptations. After 10 h, the administration of sevoflurane was stopped, the circuit was opened (non rebreathing) and the FGF was increased to 10 L/min. The Gas Man Graph shows the anesthetic tension over the course of administration in the different model compartments. After 5 h of sevoflurane administration, the inspiratory (delivered, DEL) partial pressure was set to 0 and the fresh gas flow (FGF) was increased to 10 L/min. On the bottom right corner in Fig. 1, Gas Man Graph indicates the wash-out in the different model compartments (compare Fig. 2). Please note that the partial pressure values are displayed in % of 1 Atm

Gas Man is a physiology-based computer simulation software for simulating uptake, distribution, and elimination of inhalational anesthetics. It predicts expired partial pressure during induction and awakening with high accuracy and can improve patient care [28, 29]. It contains a flow-limited four-compartment mammillary model (alveolar gas, the vessel-rich group, muscle group and fat group) of tissues, connected with the circle system. Gas Man displays graphically and numerically anesthetic partial pressures in the four compartments as they equilibrate with the anesthetic brought to them by blood flow. The volume of a distinct compartment and the anesthetic flow into the volume determine the time course of compartment equilibration. The graphical user interface and a numeric input method allow the user to change the solubility in blood and tissue groups, specific tissue fractional blood flows and specific tissue volumes and adjust vaporizer setting, FGF, alveolar ventilation (VA) and CO during the course of a simulated anesthetic administration (Fig. 1) [30]. The theoretical model is able to reproduce the kinetic relationships described by Kety and explored by Eger [12, 26, 31, 32]. Furthermore, the Gas Man simulation model has been used extensively in pharmacokinetic studies to investigate the uptake, distribution and elimination of volatile anesthetics during different clinical settings [25, 27, 33, 34].

Since there is no software solution available to simulate pharmacokinetics of inhaled anesthetics in obese patients, we created three physiology-based obese models and successively compared these to a 70 kg lean patient model. The Gas Man simulation software allows the user to independently adapt the model’s tissue volumes and relative blood flow. These adaptations performed in this study can be done by every user without additional software solutions. In future program versions, simulation models with altered physiology (i.e. obesity) will implemented.

Just increasing the weight of the model would not simulate an obese, but a big patient. Therefore, we simulated excess fat with the same flow-to-volume ratio as the fat in the 70 kg lean patient. Because flows generally scale with weight (or volume) raised to the 0.75 power [35], the fat flow in the obese models were calculated as follows in Eq. 1, [35]

1 $$\mathrm{F}\mathrm{f}\mathrm{a}{\mathrm{t}}_{\mathrm{n}\mathrm{e}\mathrm{w}}=\mathrm{F}\mathrm{f}\mathrm{a}{\mathrm{t}}_{\mathrm{o}\mathrm{l}\mathrm{d}}{\left(\frac{\mathrm{V}\mathrm{f}\mathrm{a}{\mathrm{t}}_{\mathrm{n}\mathrm{e}\mathrm{w}}}{\mathrm{V}\mathrm{f}\mathrm{a}{\mathrm{t}}_{\mathrm{o}\mathrm{l}\mathrm{d}}}\right)}^{0.75}$$

where Ffat_new_ is the adapted fat flow, Ffat_old_ the standard fat flow, Vfat_new_ the adapted fat volume and Vfat_old_ the standard fat volume (Tables 1, 2). Total CO was then calculated as the sum of the 70 kg patient’s CO and the excess fat blood flow. Fractional tissue flows for the standard 70 kg Gas Man model were then adapted to create the obese models. Since Gas Man parameters are fractional flows summed to 100%, the CO was computed as the sum of the adapted fractions of tissue flows. We computed those fractions for each patient model by successive approximation in order to cope with the nonlinearity of the fat flow- to volume-ratio relationship. Because of the increase of CO in the obese models, each simulated anesthesia in the obese models was induced with an initially increased inspiratory fraction of volatile anesthetic. That enabled an identical rise in alveolar gas partial pressure for each body weight (Table 2). To calculate the initial overpressure, we used the theoretical pharmacokinetic model of the alveolar tension curve representing the relationship between the alveolar and inspired anesthetic tension over the time [30]. When the anesthetic tension in the alveolar gas rises, the alveolar tension curve increases. After a distinct amount of time, the alveolar tension curve reaches a plateau. This so called alveolar plateau is mainly influenced by alveolar ventilation (VA), CO, and agent blood/gas partition coefficient. The equilibrium of anesthetic tension in the alveolar gas and pulmonary alveolar blood is shown in Eq. 2,

**Table 1:** Standard pharmacokinetic settings as used in Gas Man (version 4.2 [36])

	Parameter	Desflurane	Sevoflurane	Isoflurane
	1 MAC	6.0	2.1	1.1
Tissue/gas partition coefficients	Blood	0.42	0.65	1.30
	Brain	0.54	1.10	2.10
	Muscle	0.97	2.40	4.50
	Fat	13.00	34.00	70.00
Tissue/blood partition coefficients	Brain	1.29	1.69	1.11
	Muscle	2.31	3.69	2.37
	Fat	30.95	53.21	36.84

*MAC* minimal alveolar concentration

Please note that the used blood/gas partition coefficients differ from them observed by Esper et al. [54]. These pharmacokinetic values can be changed in a file in the installation directory of the Gas Man simulation software (“Drive:\Program Files (× 86)\MedMan\GasMan”). The pharmacokinetic settings used in the Gas Man simulation software are based on the work of Yasuda et al. [9]

**Table 2:** Model parameter settings

Variable		70 kg model	100 kg model	125 kg model	150 kg model
CO	(L/min)	5.00	5.47	5.73	6.03
VA	(L/min)	4.00	4.00	4.00	4.00
VRG	Volume (L)	6.00	6.00	6.00	6.00
	Flow (%)	76.00	69.53	66.30	63.06
MUS	Volume (L)	33.00	33.00	33.00	33.00
	Flow (%)	18.00	16.47	15.70	14.94
FAT	Volume (L)	14.50	44.87	62.74	81.98
	Flow (%)	6.00	14.00	18.00	22.00
	Flow (L min^−1^)	0.30	0.77	1.03	1.33
	Flow (ml min^−1^) per 100 ml of tissue	2.06	1.71	1.64	1.62
Inspired overpressure (% of 1 Atm.)	Desflurane	9.2	9.4	9.6	9.8
	Sevoflurane	3.8	4.0	4.1	4.2
	Isoflurane	2.9	3.1	3.15	3.3
Time to 1.0 MAC alveolar (min)	Desflurane	4	4	4	4
	Sevoflurane	4	4	4	4
	Isoflurane	3	3	3	3

*CO* cardiac output, *VA* alveolar ventilation, *VRG* vessel-rich tissue group compartment, *MUS* muscle tissue compartment, *FAT* fat tissue compartment, *MAC* minimal alveolar concentration

2 $$\frac{{\mathrm{P}}_{\mathrm{a}}}{{\mathrm{P}}_{\mathrm{i}}}=\frac{1}{1+\frac{\mathrm{C}\mathrm{O}\cdot\uplambda }{\mathrm{V}\mathrm{A}}}$$

where P_a_ is alveolar partial pressure, P_i_ is inspired partial pressure; λ, blood/gas partition coefficient (Table 1 [9, 36]) and VA is alveolar ventilation (Table 2).

It follows, that the alveolar plateau height increases with increasing inspired anesthetic tension, increased alveolar ventilation, decreased CO and decreased blood/gas partition coefficient [30].

During the administration of volatile anesthetics, the inspiratory and expiratory partial pressures are usually monitored continuously. Due to distribution, the expiratory anesthetic partial pressure only reflects the VRG partial pressure at steady state. It follows that, especially during induction and awakening from volatile anesthesia, the expiratory partial pressure cannot be used to estimate partial pressure at the site of action. Since we would like to perform simulations as close as possible to the clinical situation, the inspired anesthetics’ partial pressure was adapted continuously to the expired partial pressure. To ensure a consistent attainment of the desired alveolar tension in the different simulation models, the inspired volatile anesthetics’ partial pressure was adapted to the increased cardiac output, tissue volumes and flow rates. As soon as the desired partial pressure corresponding to 1.0 MAC was reached in the alveolar compartment, the vaporizer was adjusted to maintain this value.

**Fig. 2:**
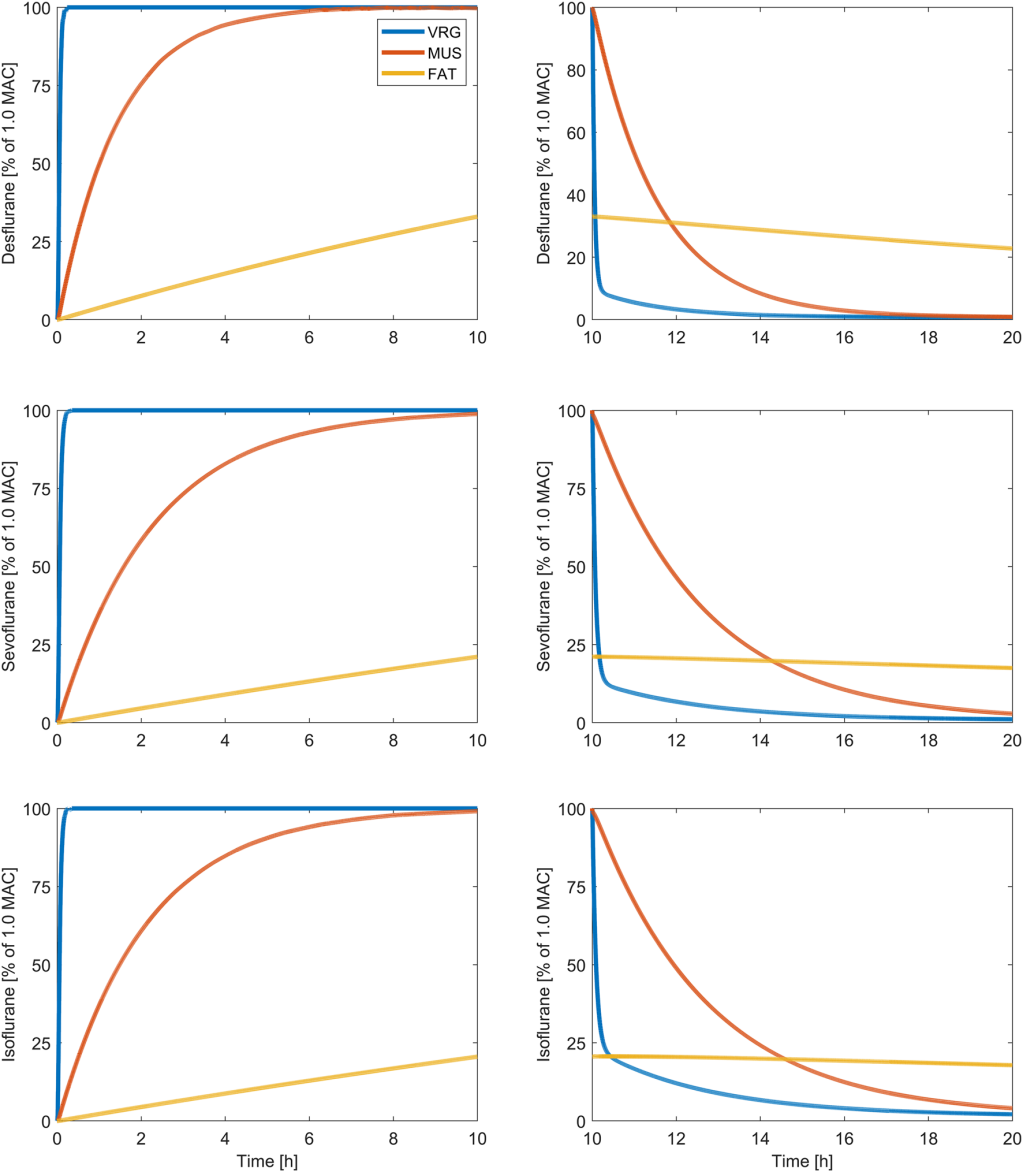
Exemplary course of anesthetic partial pressure in the vessel-rich-group (VRG), muscular (MUS) and fat (FAT) compartment. Volatile anesthesia was simulated using the validated 70 kg Gas Man simulation model. Duration of simulated volatile anesthesia was 10 h. After 10 h, the administration of sevoflurane was stopped, the circuit was opened (non rebreathing) and the FGF was increased to 10 L/min. MAC, minimal alveolar concentration. Please note that, compared to the partial pressure increase in the VRG- and MUS compartment, the increase in partial pressure in the fat compartment is very slow. This is due to the high fat/gas partition coefficients of the volatile anesthetics and the low fat perfusion

For each model (70 kg, 100 kg, 125 kg and 150 kg) and each investigated inhalational agent (desflurane, sevoflurane and isoflurane), anesthesia durations from one to nineteen hours were simulated, resulting in a total of 228 simulations. To avoid rebreathing of volatile anesthetics at the end of anesthetic administration, the breathing circuit was switched to an open (non-rebreathing) circuit, the vaporizer was set to zero, the fresh gas flow was increased to 10 L/min and the times to the anesthetic decrements by 67% (awakening), 90% (recovery) and 95% (resolution) in the vessel-rich tissue group (VRG) compartment, which reflects the highly perfused tissue of the central nervous system, were determined (Fig. 2). Hedenstierna et al. investigated dead space and VA in morbidly obese patients during mechanical ventilation. They found no significant difference in dead space ventilation and VA compared to non-obese patients [37]. Thus, VA was constant in all simulation models.

## Results

**Fig. 3:**
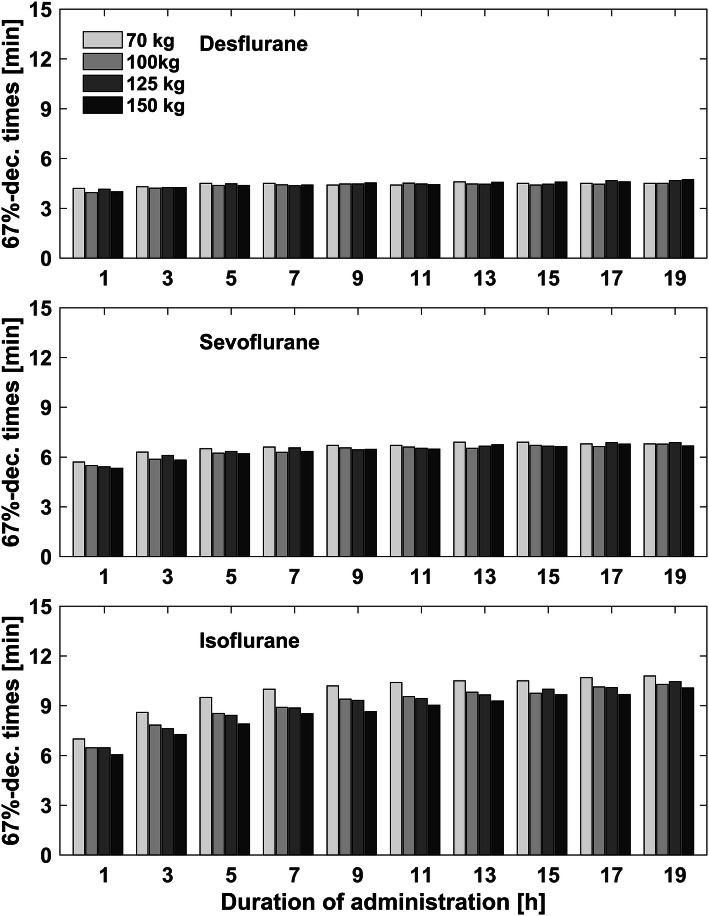
Awakening times (67%-decrement times)

**Fig. 4:**
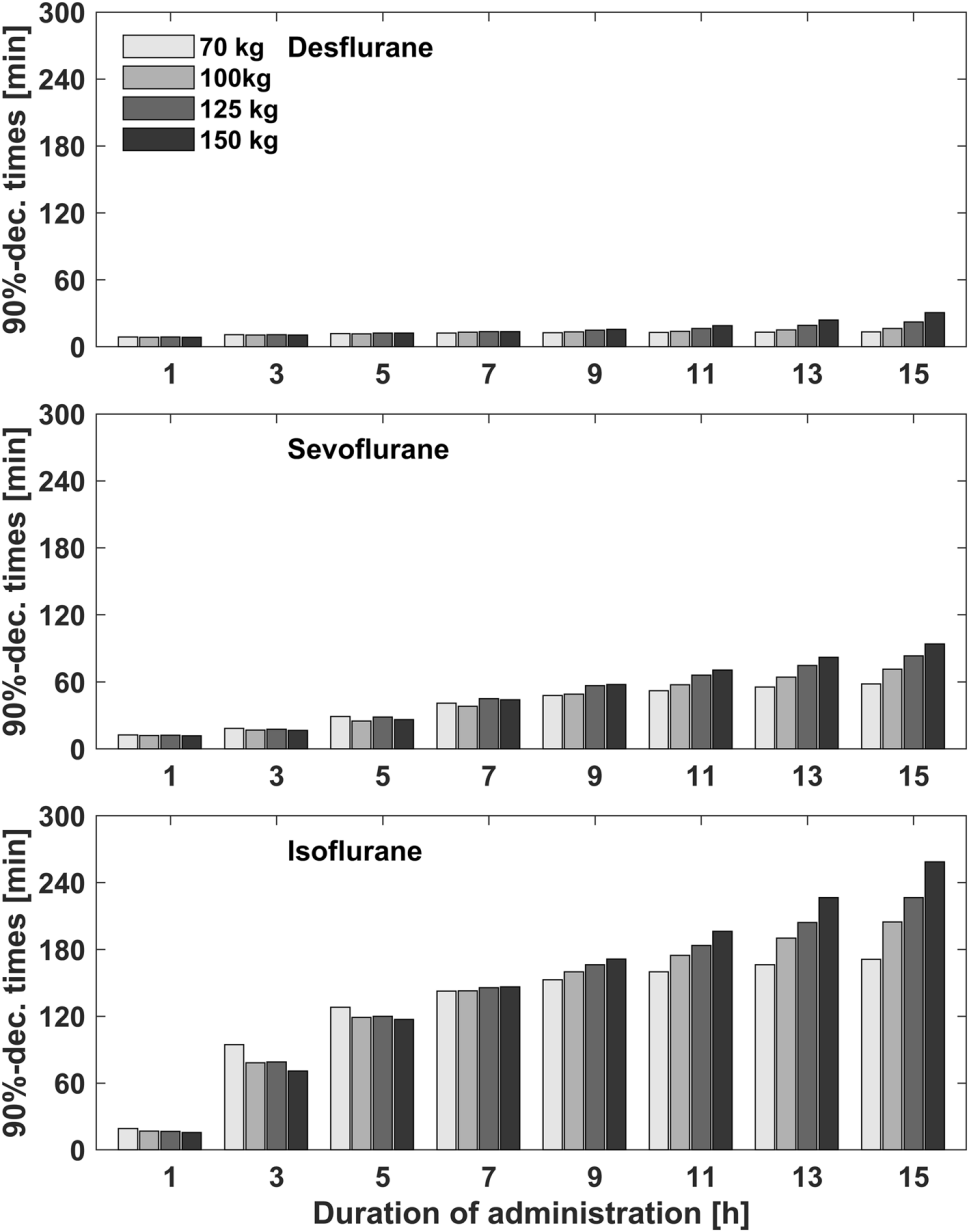
Recovery times (90%-decrement times)

**Fig. 5:**
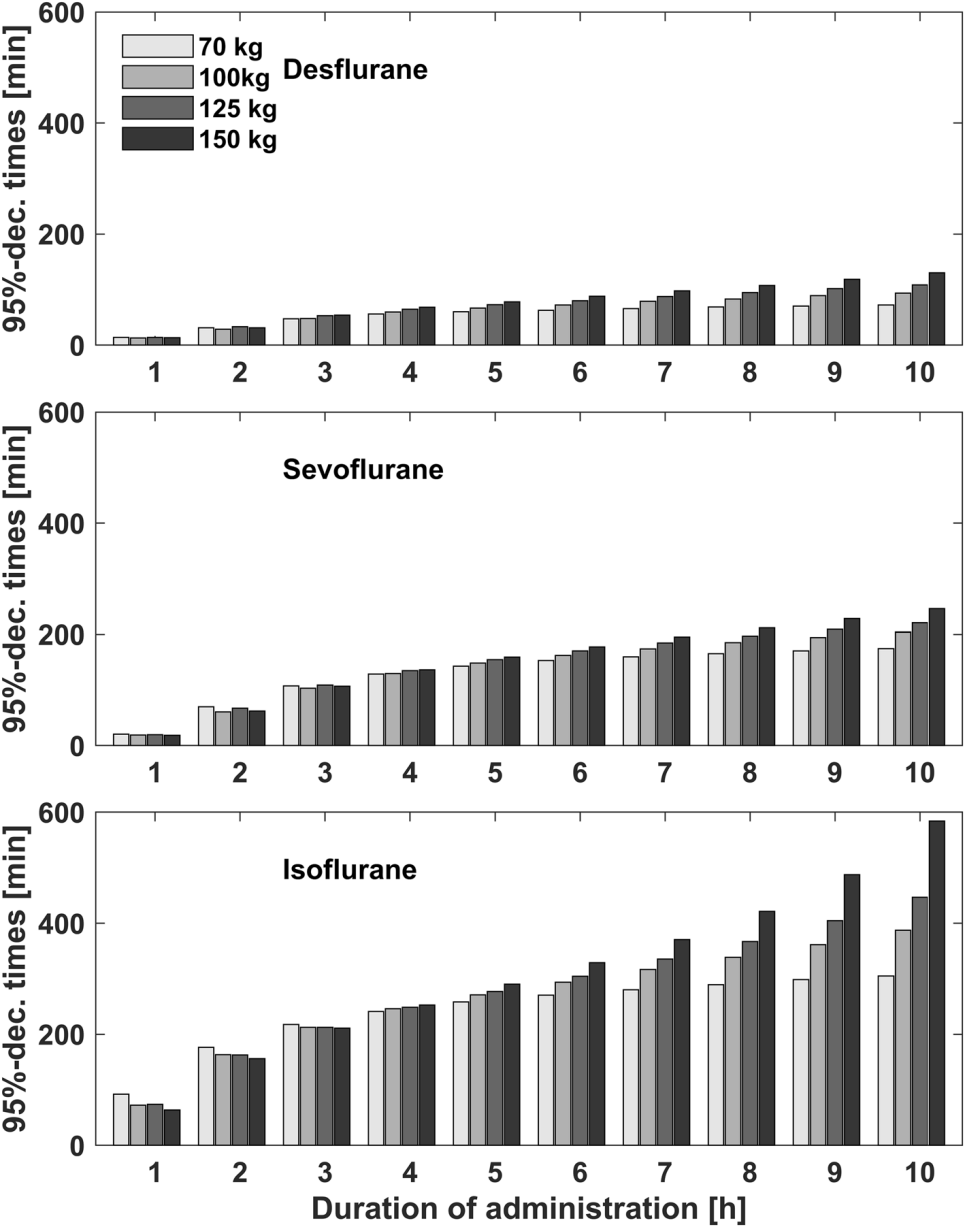
Resolution times (95%-decrement times)

With increasing duration of anesthesia, awakening times increased and lay in the range between 3 min and 57 s to 4 min and 30 s for desflurane, 5 min and 19 s to 6 min and 42 s minutes for sevoflurane and 6 min and 3 s to 10 min and 12 s for isoflurane (Table 3). For desflurane and sevoflurane this was only weakly pronounced. For isoflurane, awakening times increased by 30% with increasing anesthesia time from 1 to 19 h. The differences in awakening times between the simulation models were more obvious after volatile anesthesia with isoflurane and indicated faster elimination kinetics in the obese models (Fig. 3, Table 3). Recovery times were 8–31 min for desflurane, 11–95 min for sevoflurane and 16–260 min for isoflurane. Recovery times increased, as well as awakening times, with increasing duration of anesthesia. Recovery times increased for duration above 5 h for desflurane and, above 1 h for sevoflurane and isoflurane. After a duration of administration between 1 and 5 h, isoflurane showed longer recovery times in the obese models. Above 5 h of administration of isoflurane, the obese models reached the recovery times later than the standard 70 kg model (Fig. 4, Table 4). Resolution times lay in the range between 13 and 130 min for desflurane, 19–247 min for sevoflurane and 64 to 584 min for isoflurane. Above a duration of 1 h, resolution times increased for all three volatile agents. At administration of 5 h and more, the resolution times started to differ and indicated slower elimination kinetics in the obese simulation models (Fig. 5, Table 5). After 10 h of isoflurane administration, the resolution time for the 150 kg simulation model was above 584 min. Since Gas Man version 4.2 allows a simulation of maximal duration of 20 h, we were not able to calculate resolution times for the 150 kg model for isoflurane administration of > 10 h.

**Table 3:** Awakening times for desflurane, sevoflurane and isoflurane after a duration of anesthesia of 1, 5 and 10 h for the four different simulation models

Anesthetic	Duration of anesthesia (h)	70 kg	100 kg	125 kg	150 kg
Awakening times (min:s)					
Desflurane	1	04:12	03:57	04:09	04:00
	5	04:30	04:23	04:29	04:23
	10	04:24	04:17	04:28	04:25
Sevoflurane	1	05:42	05:29	05:25	05:19
	5	06:30	06:14	06:20	06:12
	10	06:42	06:34	06:33	06:22
Isoflurane	1	07:00	06:28	06:28	06:03
	5	09:30	08:32	08:25	07:54
	10	10:12	09:25	09:26	08:54

Decrement times are displayed in min:s. Since one simulation model only has one decrement time for one specific volatile agent and duration of administration, no further statistical analysis can be performed

**Table 4:** Recovery times for desflurane, sevoflurane and isoflurane after a duration of anesthesia of 1, 5 and 10 h for the four different simulation models

Anesthetic	Duration of anesthesia (h)	70 kg	100 kg	125 kg	150 kg
Recovery times (h:min:s)					
Desflurane	1	00:08:48	00:08:33	00:08:39	00:08:28
	5	00:11:54	00:11:36	00:12:21	00:12:15
	10	00:12:54	00:13:28	00:15:20	00:16:53
Sevoflurane	1	00:12:42	00:12:03	00:12:15	00:11:44
	5	00:29:06	00:25:02	00:28:32	00:26:17
	10	00:50:42	00:53:42	01:01:41	01:04:26
Isoflurane	1	00:19:18	00:16:51	00:16:45	00:15:36
	5	02:08:00	01:59:04	01:59:58	01:57:09
	10	02:35:54	02:48:30	02:55:12	03:03:58

Decrement times are displayed in h:min:s. Since one simulation model only has one decrement time for one specific volatile agent and duration of administration, no further statistical analysis can be performed

**Table 5:** Resolution times for desflurane, sevoflurane and isoflurane after a duration of anesthesia of 1, 5 and 10 h for the four different simulation models

Anesthetic	Duration of anesthesia (h)	70 kg	100 kg	125 kg	150 kg
Resolution times (h:min:s)					
Desflurane	1	00:14:00	00:13:02	00:13:45	00:13:18
	5	01:00:00	01:06:35	01:13:09	01:17:49
	10	01:12:24	01:33:30	01:48:30	02:10:22
Sevoflurane	1	00:20:42	00:18:56	00:19:38	00:18:38
	5	02:23:00	02:28:35	02:34:32	02:39:15
	10	02:54:30	03:23:55	03:40:52	04:06:23
Isoflurane	1	01:32:00	01:12:30	01:14:05	01:04:00
	5	04:18:12	04:30:49	04:37:18	04:50:16
	10	05:05:12	06:27:36	07:26:45	09:43:56

Decrement times are displayed in h:min:s. Since one simulation model only has one decrement time for one specific volatile agent and duration of administration, no further statistical analysis can be performed

## Discussion

This is the first study to create obese simulation models and to conduct pharmacokinetic calculations of volatile anesthetic elimination for these obese simulation models using the Gas Man simulation software. Our simulations validate and enlarge earlier findings for the context-sensitive decrement times of Eger et al. and Bailey et al. [12, 26, 38] and indicate that in most clinical situations the increase in fat volume may only minimally affects the awakening times after volatile anesthesia and that the blood/gas partition coefficient is the primary determinant of the awakening times [12, 26].

Due to their beneficial physicochemical properties, desflurane and sevoflurane are known to produce a faster awakening profile in morbidly obese patients compared with isoflurane [6, 9, 13, 15, 39]. In a clinical study, Casati et al. examined the effect of obesity on the onset and offset of desflurane and sevoflurane. They measured the actual end-tidal partial pressure (F_A_) to last measured end-tidal fraction before offset (F_A0_) of sevoflurane in normal weight and obese patients. During the elimination process, elimination of volatile anesthetics was characterized by a decrease of the F_A_/F_A0_, quotient. F_A_/F_A0_ decreased slower for sevoflurane in the obese patients [11]. Similar elimination kinetics of sevoflurane were described by La Colla et al. in morbidly obese patients and indicated faster elimination of desflurane compared to sevoflurane after volatile anesthesia [14]. A meta-analysis performed by Singh et al. showed that obese patients recover faster from volatile anesthesia with desflurane compared to sevoflurane [40]. Further, the time to extubation and patient responsiveness was shorter after volatile anesthesia with desflurane than with sevoflurane [40]. Identical trends in recovery profiles of desflurane and sevoflurane were reported earlier in a meta-analysis for non-obese patients [41]. In a randomized clinical trial, Strum et al. also compared awakening and recovery profiles of desflurane and sevoflurane after volatile anesthesia in obese patients. Although obese patients in this clinical trial were anesthetized longer with desflurane than with sevoflurane, significantly earlier recovery of response and tracheal extubation occurred with desflurane [16].

Data regarding the influence of obesity on the context-sensitive decrement times after longer administration (e.g. 10 h) are limited and there are no clinical investigations comparing the recovery of obese and morbidly obese patients after longer durations of volatile anesthesia. Further, there are conflicting data regarding recovery in obese patients. De Baerdemaeker et al. compared the post anesthesia recovery in morbidly obese patients (mean body mass index in desflurane group of 41 [5] and sevoflurane group 41 [6] kg m^2^) after volatile anesthesia combined with a target controlled infusion of remifentanil (duration of administration in desflurane group of 112 [28] and sevoflurane group 112 [33] minutes) after laparoscopic gastric banding [42]. In this clinical investigation, postoperative recovery was assessed with the Aldrete score [43]. They found that the overall postoperative recovery did not differ between the two anesthetic groups. In regard to this clinical trial and the results of our simulations, one might speculate that the short duration of administration did not enable higher anesthetic saturation in the excess fat tissue compartment. One other very important issue is the concept of the MAC-awake (the expiratory partial pressure of volatile anesthetic to suppress the response to a verbal command in 50% of the patients) that we used to calculate awakening times. For isoflurane and desflurane, it was one-third of 1.0 MAC. The calculated 67% decrement times for isoflurane and desflurane showed no significant differences between the simulation models, but the threshold of potentially measurable disturbance of cognition equaled 0.1 MAC [44, 45]. To describe and characterize a decrement of partial pressure in the patient’s brain, to get to a distinct discussion and do clinical predictions, we should observe the 95% decrement times. When our simulation model reaches the resolution threshold in the VRG, the central nervous partial pressure would be 0.05 MAC. In this regard it should be noted that other anesthetic drugs are known to lower the anesthetic requirement to achieve a certain clinical anesthetic effect [46, 47].

Levitt et al. used a physiologically based pharmacokinetic model to describe the long-term kinetics of volatile anesthetics, earlier described by Yasuda et al. [7, 9, 48]. In accordance with the results of our simulation study, they demonstrated that during the first 3 h of anesthesia the adipose tissue absorbs the volatile anesthetics. For this period of time, the heterogeneity of adipose tissue blood flow (ATBF) was not important and the adipose perfusion of 0.044 L min^−1^ kg^−1^ yielded the best model fit. Above this duration of application, the heterogeneity of ATBF was modeled into less and more perfused fat compartments (0.014 and 0.074 L min ^−1^ kg^−1^) [48]. This model provides a good fit as compared to data of the pharmacokinetics of cannabinol and shows direct qualitative evidence that there is no significant adipose tissue diffusion limitation. Eger et al. suggested that this separation into a less and more perfused adipose tissue compartment might be an artifact of inter-tissue diffusion from well perfused organs (e.g. kidney, dermis) and adjective fat tissue. However, this possible explanation does not seem to be consistent with the pharmacokinetic study results of Ohlsson et al. and Levitt et al. on the lipophilic cannabinoids [48, 49].

Cork et al. tried to prove the concept of fat-solubility on the kinetics of intravenous and volatile anesthetics. Their data suggested that the fat/gas partition coefficients does not influence the awakening and discharge times from the operating room in morbidly obese patients [29]. Torri et al. showed that sevoflurane provides a faster awakening, recovery and earlier discharge from the operating room than isoflurane in morbidly obese patients undergoing bariatric surgery [50]. Even if the fat/blood-partition coefficients of sevoflurane and isoflurane are almost identical, the big difference in blood/gas partition coefficient may explain the difference in recovery and resolution in the obese simulation models.

These Gas Man simulations and decrement time calculations show that a pharmacokinetic simulation reflects and details the pathophysiological changes of uptake, distribution and most importantly on elimination of volatile anesthetics in obese patients. In addition to previous theoretical investigations by Eger et al. [12], adipose tissue might even accelerate awakening by acting as a buffer for volatile anesthetics. This observation is more pronounced after shorter volatile anesthesia and for volatile anesthetics with higher tissue/gas partition coefficients (comp. Fig. 3, Table 1). The direct comparison between the different decrement times in the most obese model (150 kg, comp. Table 1) suggest desflurane as the agent of choice for anesthesia in obese models. Particularly in longer anesthesia and in combination with other, MAC-reducing anesthetic drugs, prolonged recovery and postoperative complications can be avoided by choosing less soluble inhalation agents like sevoflurane and desflurane. When shorter anesthesia is performed, the excess body fat seems to be an expansion reservoir for inhaled anesthetics. This finding is consistent with earlier calculations by Yasuda et al., Levitt et al. and Eger et al. [7, 9, 12, 26, 32, 48]. It could also be one possible explanation for the faster 90% isoflurane-decrement in the obese model between a duration of anesthesia from 1 to 6 h (Fig. 4). These findings suggest that the fat/blood partition coefficient does not, at least clinically, influence the recovery times for volatile anesthetics in morbidly obese patients. Furthermore, they underline the lack of importance of anesthetic accumulation in fat tissue on awakening times for the less soluble agents desflurane and sevoflurane. Since elimination kinetics are only minimally affected by the increase in fat tissue in our obese simulation models, the predictions by anesthesia decision support tools like SmartPilot® (Dräger Medical) and Navigator® (GE Healthcare) may be suited for obese patients. In obese patients, the uptake of volatile anesthetics, especially of those with higher blood-gas partition coefficients, is increased [15]. This increased uptake can also be observed in our obese simulation models. To ensure a comparable fast rise of the expiratory concentration in the non-obese and the obese simulation models, we adapted the inspiratory concentration during induction. It should be verified clinically, if the existing end-tidal control delivery systems like Zeus (Dräger), Aisys ETC (GE Healthcare) and Flow-I (Maquet) are able to do so as well. It follows that the obese simulation models of this study might be useful to refine those end-tidal control delivery systems or that the end-tidal control delivery performance might be useful to refine the model for obese patients and enhance our understanding of the obesity-fat impact on kinetics.

## Limitations

We know that subcutaneous fat volume dominates over internal fat volume in obese humans and that density of normal and obesity human fat is approximately 0.9 g mL^−1^ [51]. Compared to lean body fat, it is likely that obese fat is perfused relatively poor [17]. But, to assess the exact fat volume and heterogeneous fat tissue flows is difficult [52]. We also know that inter-tissue diffusion impacts fat distribution of many substances [17]. Because of the heterogeneity of fat perfusion and the fact that this inter-tissue diffusion is not modelled in Gas Man [36], we chose a non-linear fat perfusion model. Further, we did not find any evidence that the allometric scaling we used to calculate the fat perfusion in our obese simulation models is physiologically comprehensible. One might argue that this allometric scaling is not justified in the case of an increase in tissue volume, as it would result in a relative underestimated tissue flow. However, if the relative fat perfusion would increase linearly with fat volume, the equilibration between the blood and fat compartment would have been faster. It follows that a higher fat perfusion would have increased the recovery and resolution times in the obese simulation models.

Further it should be noted that because of the negative impact of the excessive adipose tissue on the respiratory system mechanics, obese patients are prone to develop atelectasis during mechanical ventilation [53]. Since the impact on the impaired respiratory mechanics and atelectasis on alveolar ventilation in obese patients depends on many factors (e.g. patient positioning, duration of ventilation, surgical procedure) we did not alter alveolar ventilation in our obese simulation models. Others may want to explore the effect of obesity on the kinetic effects of respiratory variation.

Our simulation model can also be connected with a reduction or increase in muscle tissue [52]. Likewise to the fat volume and relative perfusion in obese humans, the alteration in muscle mass and muscle perfusion is also subject to large variations [52]. Hence, we did not change the muscle volume in our simulations. Others may want to explore these effects in the future.

## Conclusions

This is the first study to investigate the influence of different obesity related model adaptions of the Gas Man simulation model on context-sensitive decrement times for volatile anesthetics after durations of administration between 1 and 19 h. The main finding is that in our obese simulation models, awakening times are nor prolonged and that the Gas Man simulation software can be used to simulate the theoretically observed effects of obesity on the pharmacokinetic of volatile anesthetics by Eger et al. [12]. After simulated volatile anesthesia with sevoflurane and isoflurane, the additional adipose tissue seems to act more like a storage for volatile anesthetics and thus shortens awakening times. After short duration of administration, the volatile anesthetics’ partial pressure in less perfused body compartments (e.g. fat tissue) is low and the excessive adipose tissue may act more like a sink than like a source of anesthetic.

Desflurane showed the overall fastest kinetics in our obese simulation models. Since this is the first pharmacokinetic modelling of adipose simulation models with Gas Man, the results should be validated clinically and it should be stressed that these simulations cannot provide advices to clinical management and drug administration. However, as part of modern individualized drug titration strategies, pharmacokinetic models may be implemented into clinical monitoring to estimate the volatile anesthetic partial pressure in the central nervous system to help predict anesthesia awakening time.
